# Newly Characterized Region of CP190 Associates with Microtubules and Mediates Proper Spindle Morphology in *Drosophila* Stem Cells

**DOI:** 10.1371/journal.pone.0144174

**Published:** 2015-12-09

**Authors:** Karen M. Plevock, Brian J. Galletta, Kevin C. Slep, Nasser M. Rusan

**Affiliations:** 1 Cell Biology and Physiology Center, National Heart, Lung, and Blood Institute, National Institutes of Health, Bethesda, Maryland, 20892, United State of America; 2 Department of Biochemistry and Biophysics, University of North Carolina, Chapel Hill, North Carolina, 27599, United States of America; 3 Department of Biology, University of North Carolina, Chapel Hill, North Carolina, 27599, United States of America; Institut de Génétique et Développement de Rennes, FRANCE

## Abstract

CP190 is a large, multi-domain protein, first identified as a centrosome protein with oscillatory localization over the course of the cell cycle. During interphase it has a well-established role within the nucleus as a chromatin insulator. Upon nuclear envelope breakdown, there is a striking redistribution of CP190 to centrosomes and the mitotic spindle, in addition to the population at chromosomes. Here, we investigate CP190 in detail by performing domain analysis in cultured *Drosophila* S2 cells combined with protein structure determination by X-ray crystallography, *in vitro* biochemical characterization, and *in vivo* fixed and live imaging of *cp190* mutant flies. Our analysis of CP190 identifies a novel N-terminal centrosome and microtubule (MT) targeting region, sufficient for spindle localization. This region consists of a highly conserved BTB domain and a linker region that serves as the MT binding domain. We present the 2.5 Å resolution structure of the CP190 N-terminal 126 amino acids, which adopts a canonical BTB domain fold and exists as a stable dimer in solution. The ability of the linker region to robustly localize to MTs requires BTB domain-mediated dimerization. Deletion of the linker region using CRISPR significantly alters spindle morphology and leads to DNA segregation errors in the developing *Drosophila* brain neuroblasts. Collectively, we highlight a multivalent MT-binding architecture in CP190, which confers distinct subcellular cytoskeletal localization and function during mitosis.

## Introduction

The MT cytoskeleton is a dynamic polymer, essential for many intracellular processes including cell structure, cell migration, MT motor-based intracellular transport, and mitosis. Each of these MT functions requires a dynamic MT network. While MTs do exhibit dynamic instability *in vitro* [1,2], the MT network is regulated spatially and temporally by a host of MT-associated proteins (MAPs) *in vivo*. MAPs modulate MT dynamics by altering the rates of polymerization (growth); de-polymerization (shrinkage); or the frequency of MT pause, catastrophe, or rescue. They can also crosslink adjacent MTs and/or link MTs to other subcellular structures and organelles, and establish local MT network polarity; e.g. linking MT minus ends to a centrosome [3–7]. During mitosis, MAPs play critical roles driving restructuring of the MT network into a highly coordinated, dynamic bipolar spindle. MAPs in mitosis performing various functions are located at kinetochores, throughout the mitotic spindle, and at centrosomes, the non-membrane bound organelles that organize mitotic spindle poles. Centrosomes include a core pair of centrioles surrounded by MT-nucleating γ-Tubulin Ring Complexes (γTuRCs) embedded in pericentriolar material [PCM; 4]. Determining the molecular composition of the PCM, including MAPs, and investigating the cell cycle-dependent molecular function of these components is a major focus of centrosome biology research.

**Fig 1:**
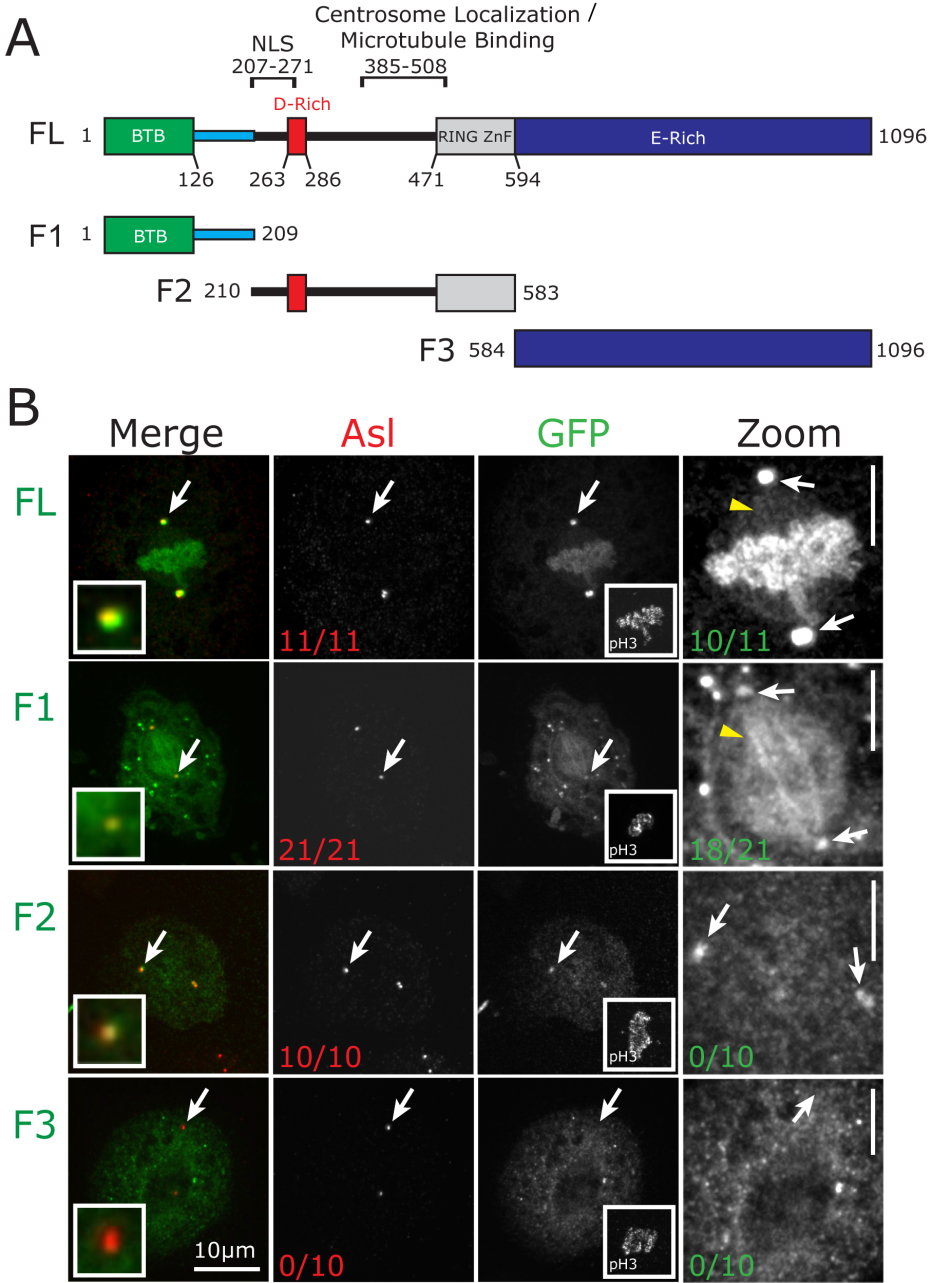
CP190 localization to the mitotic spindle is driven by its N-terminal region. *A*, Domain structure of CP190. Shown is the BTB domain (green), linker (blue), D-rich domain (red), Zinc finger domain (grey), and the E-rich domain (blue). Indicated above the graphic representation are the previously identified nuclear localization signal (NLS) and the centrosome localization/MT binding domain [11]. Below the full length (FL) CP190 is a schematic of fragments used for this study (F1, F2, and F3). *B*, *Drosophila* S2 cells transfected with the indicated GFP-CP190 constructs (green). Shown are mitotic cells fixed and stained for Asterless (Asl, red) to mark centrosomes and pH3 (mitotic specific histone marker, inset in the GFP column). White arrows designate the centrosome. Red numbers on Asl column indicate the fraction of mitotic cells that exhibit GFP localization to centrosomes. Zoom of GFP channel (right column) is contrast enhanced to emphasize GFP signal on the mitotic spindle (yellow arrowheads). Green numbers indicate the fraction of mitotic cells with GFP signal at the spindle. Scale bars (B) = 10 μm, (zoom) = 5 μm.

The *Drosophila melanogaster* centrosome associated protein at 190 kDa (CP190) was first identified as a MAP using MT affinity chromatography [8]. After localizing it to centrosomes, subsequent studies used antibodies against CP190 as bait to identify additional centrosome proteins [9]. Notably, CP190 was found within a cytoplasmic scaffolding complex that includes the centrosomal proteins Sas-4, Asterless, Centrosomin, Pericentrin-Like protein, and γ-tubulin [10]. CP190 exhibits prominent cell cycle oscillatory localization [11,12]. During mitosis, CP190 localizes to centrosomes and the mitotic spindle. In contrast, interphase CP190 localizes to the nucleus where it functions in three key chromatin insulator complexes organized by Su(Hw), BEAF32, and CTCF that collectively function to modulate gene activity [13–16]. Although CP190 insulator function has been characterized at biochemical, cellular, and organismal levels [17–20], little has been elucidated regarding its mitotic functions at centrosomes and MTs. CP190 has a complex molecular architecture that includes an N-terminal Broad-complex, Tramtrack and Bric à brac (BTB) domain, a D-rich domain, a central region with MT binding and centrosome targeting ability, and a C-terminal E-rich domain (Fig 1*A*). Specific domains in CP190 were identified that mediate localization to, and interaction with, centrosomes, MTs, and the nucleus [11]. Subsequent studies assumed that the central centrosome and MT interacting domains were the sole part of the protein competent for interaction with cytoskeletal components [21–23]. As a consequence, structure-function studies concluded that the CP190 centrosome and MT localization domain is dispensable for function [21,22]. Specifically, it was found that expressing a CP190 allele lacking the central MT and centrosome localization domain did not show MT or centrosome defects, and rescued the lethality associated with CP190 loss [22]. Further studies have shown that the CP190 BTB domain is essential for chromatin association and survival [23].

Here, we delineate a novel centrosome- and MT-interaction region in CP190, which we show requires BTB domain-mediated dimerization to properly associate with MTs. We present the structure of the CP190 homodimeric BTB domain and confirm its dimeric state in solution. Furthermore, deletion of this newly identified MT-targeting region using CRISPR/Cas9 technology results in severe spindle formation and DNA segregation defects in central brain neuroblasts (NBs). These results are the first to assign a role for CP190 in regulating MTs.

## Methods and Materials

### CP190 S2 expression constructs

We used the Gateway cloning system (Life Technologies) to generate all CP190 constructs. CP190 fragments were PCR-amplified and cloned into pENTR/D, then shuttled into a pAGW destination vector (Life Technologies). Mutations in Fragment 1 (aa 1–209) of CP190 were generated using the Quikchange (Agilent Technologies) method with KOD-Xtreme hot start DNA polymerase. Primers used for this study are listed in Table 1. Cells were transfected using Cell Line Nucleofector Kit V (Lonza Inc.) and imaged 48–96 hours later. *Drosophila* S2 cells were passaged in SF900 media supplemented with penicillin/streptomycin mix (Invitrogen) and imaged in Schneider’s media (Gibco by LifeTechnologies, Grand Island, NY) supplemented with penicillin/streptomycin mix and 5% FBS.

**Table 1:** Primers used for amplifying CP190 and generating CRISPR fly.

CP190 1F (and BTB-F)	CACCATGGGTGAAGTCAAGTCCGTGAAAGTG
CP190 1R (and 1L-R)	tggctcctgcttcacattgctactatc
CP190 1L-F	CACC ATG cctagtccaaagggaa
CP190 BTB-R	CGGCCTTTGCTGGCGATTAACGTTCTC
CP190 2F	CACCATGacgtcaccattcgagcagctgcgaaag
CP190 2R	ctgctccttgtggtagctcttcatgtg
CP190 3F	CACCATGgctttggaggatggcattatcgatgaaac
CP190 3R	tagctcctccttcgccgccgcactaac
L20E F	CTTCTTCCTGCAGAAGGAGCAGAACTTCTTTAATAAAAC
L20E R	GTTTTATTAAAGAAGTTCTGCTCCTTCTGCAGGAAGAAG
DSRNA- F	GTACGTAATACGACTCACTATAGGGAGCCGCGAGATGACATTAGT
DSRNA- R	GTACGTAATACGACTCACTATAGGGGAATGCGGAATTGGTGAATC
3’UTR DSRNA-F	GTACGTAATACGACTCACTATAGGGCAGCAGATAAACGCACCTGA
3’UTR DSRNA-R	TACGTAATACGACTCACTATAGGGCATGCTAGCAGGGCAACATA
CP190 pENTR gibsonR	TGATCGCTCAGGGAGCAGAGAATACTACTGctagacAAGGGTGGGCGCGCCGACCCAGCT
CP190 pENTR gibsonF	GCAGGCTCCGCGGCCGCCCCCTTCACCaggTTTCGCGCCGTGGCGGCAGAGCAAAATAAA
Seam ΔLinker F	tattttgtaaccttttattttctttagCCGACGTCACCATTCGAGCAGCTGCGAAAGGGT
Seam ΔLinker R	ACCCTTTCGCAGCTGCTCGAATGGTGACGTCGGctaaagaaaataaaaggttacaaAata
CP190 Check F	CGGGACAATTCACAGCTAAAGGTAC
Mutate PAM F	GTGCTGTTGAAGCTGCTAGAAGCGCACCGTCGCACCATGG
Mutant PAM R	CCATGGTGCGACGGTGCGCTTCTAGCAGCTTCAACAGCAC
Guide F	cttcGGTGCTGTTGAAGCTGCTAG
Guide R	aaacCTAGCAGCTTCAACAGCACC

### CP190 knockdown

Double-stranded RNA was generated using a CP190 C-terminal exon corresponding to amino acids 786–924 and a 3’UTR region as templates (primers used are presented in Table 1). Template was amplified from the DNA and the T7 Ribomax *in vitro* transcription kit (Promega) was used to produce double strand RNA (dsRNA). Each dsRNA was added to SF900 media at 10 μg/mL in final concentration. dsRNA was added to cells at day 0 after transfection and 2 days post-transfection. Cells were imaged on day 4. Knockdown was confirmed by western blot. Primary antibodies used include anti-CP190 antibody [14] and anti-α-tubulin (1:500, clone DM1A; Sigma-Aldrich).

### Preparing cells for imaging

S2 cells were plated on concanavalin A-coated MatTek (Ashland, MA) dishes (for live cell imaging) or #1.5 coverslips (for fixed-cell imaging) and allowed to adhere for 30 minutes. Samples to be fixed were washed in 1X phosphate-buffered saline (PBS) then fixed with -20°C 100% MeOH for 15 minutes. Samples were stained at room temperature with primary antibodies in PBS+5% Normal Goat Serum (NGS) for 1 hour and with secondary antibodies in PBS+5% NGS for 30 minutes. Primary antibodies used were guinea pig anti-Asterless (1:30,000; G. Rogers, University of Arizona Cancer Center, University of Arizona, Tucson, AZ), anti-α-tubulin (1:500, clone DM1A; Sigma-Aldrich). Secondary antibodies were Alexa Fluor 568 or 647 (1:1000; Life Technologies). Coverslips were mounted using Aqua Poly/Mount (Polysciences, Inc., Warrington, PA) [24].

### Image acquisition and analysis

Imaging was performed on a Nikon Ti Microscope using a 100X (1.49 NA) objective, a CSU-22 spinning disk confocal head (Yokogawa, Tokyo, Japan), a charge-coupled device camera (CoolSNAP HQ2; Photometrics, Tuscon, AZ) and solid state lasers (VisiTech International, Sunderland, UK). Emission filters (Semrock, Rochester, NY) at 405, 491,561, and 647 nm used for emission were controlled by a MAC6000 (Ludl Electronic Products, Hawthorne, NY). The microscope was controlled by MetaMorph software (Molecular Devices, Sunnyvale, CA). Z-stacks of acquired images were scored for co-localization of CP190 fragments with either Asl (centrosome) or α-tubulin (microtubules). Data were plotted and statistical analyses were performed using Prism6 (GraphPad Software, Inc.).

### Cloning and expression for crystallization and biochemistry

DNA encoding the *Drosophila melanogaster* CP190 BTB domain (residues 1–135) was subcloned into pET28 (Novagen) using NdeI and EcoRI restriction enzyme sites, generating a thrombin-cleavable N-terminal His_6_ tag. The pET28-CP190^BTB^ construct was transformed into BL21 DE3 (pLysS) *E*. *coli* and grown at 37°C in 6 L Luria Broth under kanamycin selection (20 μg/L) to an optical density of 1.0 (λ = 600 nm). Protein expression was induced with 100 μM IPTG for 16 hours at 20°C. Cells were harvested by centrifugation at 2100 x g for 10 min, resuspended in 200 mL buffer A (25 mM Tris pH 8.0, 300 mM NaCl, 10 mM imidazole, 0.1% β-ME) and stored at -20°C. Selenomethionine-substituted CP190^BTB^ was generated using B834 auxotrophic *E*. *coli* and minimal media containing L-selenomethionine [25].

### Protein purification

The CP190^BTB^ construct was purified by sequential Ni^2+^-NTA and ion exchange chromatography as follows. Cell pellets were thawed and lysed by sonication at 4°C. Phenylmethylsulfonyl fluoride (1 mM final) was added during lysis to prevent proteolytic degradation. Cell lysate was clarified by centrifugation at 23,000 x g for 45 min and the supernatant was loaded onto a 15 ml Ni^2+^-NTA column (Qiagen). The column was washed with 500 ml buffer A and protein was eluted using a 250 ml linear gradient between buffer A and B (buffer B = buffer A supplemented with 290 mM Imidazole). Fractions containing His_6_-CP190^BTB^ were pooled and CaCl_2_ was added to a final concentration of 1 mM. The N-terminal His_6_-tag was removed by digestion with 0.1 mg bovine α-thrombin (HTI, Essex Junction, VT) for 16 hours at 4°C while dialyzing against 4 L buffer C (buffer C = 25 mM Tris pH 9.0, 0.1% β-ME) using 3,000 MWCO dialysis tubing (ThemoScientific, Rockford, IL). Digested protein was removed from the dialysis tubing, diluted three-fold in buffer C and loaded onto a 15 ml Q-sepharose fast flow column (GE Healthcare), washed with 200 ml buffer C and eluted over a 250 ml linear gradient between buffer C and D (buffer D = buffer C supplemented with 1 M NaCl). Protein fractions containing CP190^BTB^ were pooled, exchanged into 25 mM Tris pH 8.5, 200 mM NaCl, 0.1% β-ME and concentrated to 100 mg/ml using a Millipore Ultrafree 3,000 MWCO concentrator (EMD Millipore, Darmstadt, Germany). Concentrated CP190^BTB^ protein was aliquoted, snap-frozen in liquid nitrogen, and stored at -80°C. Purification of selenomethionine-subsituted CP190^BTB^ protein proceeded according to the native purification scheme.

### Crystallization

CP190^BTB^ was crystallized using the hanging drop vapor diffusion method. 2 μl of CP190^BTB^ (native and selenomethionine-substituted protein) at 15 mg/ml was added to an equal volume of a mother liquor containing 20% PEG 3350, 160 mM ammonium citrate dibasic and equilibrated against 1 ml of mother liquor at 20°C. Crystals were transferred to LV CryoOil (MiTeGen) and flash frozen in liquid nitrogen.

### Data collection, structure determination, and refinement

Isomorphous CP190^BTB^ native and selenium single wavelength anomalous dispersion (SAD) peak data sets were collected on single crystals to a resolution of 2.5 and 2.7 Å respectively. Diffraction data was collected at the Advanced Photon Source 22-ID beamline at 100 K. CP190^BTB^ crystals belong to the space group P3_2_21 with one molecule in the asymmetric unit and had no evidence of twinning. Data was indexed, integrated, and scaled using HKL2000 [26]. Selenium sites were identified and used to generate initial experimental, density modified electron density maps (PHENIX) [27]. Initial models were built using AutoBuild (PHENIX) followed by reiterative manual building in Coot and refinement using phenix.refine (PHENIX) [27,28]. The selenomethionine-substituted structure was refined against a MLHL target function. Selenomethionines in the resulting model were changed to methionines and the resulting structure used as an initial model for the wild-type BTB domain structure. The structure was then refined against the native data to 2.5 Å resolution against a ML target function. Refinement was monitored using a Free R, using 10% of the data randomly excluded from refinement. Information regarding data statistics, model building and refinement can be found in Table 2. Atomic coordinates have been deposited in the Protein Data Bank under accession code 5EUP.

**Table 2:** Crystallographic data, phasing, and refinement statistics.

Structure	CP190 BTB Domain	
Crystal	Native	SeMet
Space Group	P3_2_21	P3_2_21
Unit Cell: *a*, *b*, *c* (Å) */* α, β, γ (°)	86.2, 86.2, 40.2 / 90, 90, 120	85.2, 85.2, 40.4 / 90, 90, 120
Wavelength (Å)	0.98038	0.98037
d_min_ (Å)	2.5 (2.59–2.50)	2.7 (2.80–2.70)
No. observations: measured / unique	32572 (3220) / 6124 (586)	103140 (9710) / 9255 (916)
Redundancy	5.3 (5.5)	11.1 (10.6)
Completeness (%)	99.5 (100.0)	100.0 (100.0)
I/σ	23.3 (3.3)	24.8 (3.2)
R_sym_ (%)	5.8 (51.1)	10.5 (87.1)
Figure of merit Centrics / Acentrics		0.32 (0.34) / 0.61 (0.48)
Refinement (Å)	50–2.5 (2.75–2.50)	
R value	0.219 (0.317)	
R_free_	0.259 (0.369)	
Rmsd bond lengths (Å)	0.002	
Rmsd bond angles (°)	0.550	
Ramachandran Plot: Favored/Allowed/Outliers (%)	97.5/2.5/0	
MolProbity Clashscore/Overall Score:	4.08/1.30	

Values in parentheses are for the highest resolution shell.

### Size-Exclusion Chromatography with Multi-Angle Light Scattering (SEC-MALS)

Protein was injected onto a Superdex 200 10/300 GL size exclusion column (GE Healthcare) pre-equilibrated in running buffer (25mM Tris pH 7.4, 0.2 g/L NaN_2_) at a flow rate of 0.5 mL/min, then passed through an in-line UV detector, a Wyatt DAWN HELIOS II light scattering instrument, and a Wyatt Optilab rEX refractometer (Wyatt Technology). Data were processed with ASTRA software and plotted using Prism6 (GraphPad Software, Inc.).

### Microtubule co-sedimentation assays

Taxol stabilized MTs were prepared and sedimentation assays conducted as described [29]. Briefly, MTs and protein were incubated at room temperature for 20 min. The reaction mixture was then layered on top of a 40% glycerol cushion and centrifuged at 50,000xg for 30 min at room temperature. A supernatant fraction was collected from the top of the sample and the pellet fraction was collected from below the glycerol cushion. Supernatant and pellet samples were analyzed by SDS-PAGE to assay for co-sedimentation.

### Generating CRISPR *cp190* ^*ΔL*^ fly

CRISPR PAM site was determined using http://tools.flycrispr.molbio.wisc.edu/targetFinder/. After identifying the target site, two complementary primers corresponding to the guide site (without the PAM) were annealed together then ligated into the U6 plasmid to make a U6-chiRNA plasmid. A repair construct (with PAM site mutated) was designed to delete the linker region at the endogenous locus using 5’ and 3’ homology arms flanking 1.1 Kb upstream and downstream of the deletion site (S6*A* Fig). Primers used are listed in Table 1. Cas9 flies were injected at BestGene Inc. (Chino Hills, CA). Single flies were selected and balanced over TM6B. Genomic preps on single fly lines were completed as previously described [30]. PCR screening and sequencing individual lines confirmed deletion of the linker region (S6*B* Fig).

### Immunofluorescence of fly central nervous system

Brains were dissected from third instar larvae from *cp190* ^*ΔL*^ and WT control flies. Brains were then fixed in 9% PFA in PBST for 20 minutes at room temperature, washed 3X10 minutes in PBST and then stained with primary antibody. Guinea pig α-Asl (1:30,000; G. Rogers, University of Arizona Cancer Center, University of Arizona, Tucson, AZ) and rabbit α-CP190 (1:1000; [14]) overnight at 4°C. Brains were then washed 3X10 minutes and treated with guinea α-guinea-pig-568 (1:500), α-Rabbit-647 (1:500) and DAPI (1:1000) for 4 hours at room temperature. After 3X10 minutes wash, brains were mounted in Aqua Poly/Mount (Polysciences, Inc., Warrington, PA) [24].

### Live imaging of MTs in CP190ΔL fly

Ubi-GFP:tubulin was introduced into *cp190* ^*ΔL*^/TM6 and *Df* ^*p11*^/TM6 flies. These two flies were then crossed to produce ubi-GFP::Tubulin;*cp190* ^*ΔL*^ */ Df* ^*p11*^ experimental flies. Larvae with the TM6 balancer were used as controls. These brains were dissected from third instar larvae and imaged as described previously [31].

## Results

### CP190’s N-terminal region contains a previously unidentified MT and centrosome-targeting domain

Previous work identified a central region of CP190 (aa 385–508) as the centrosome and MT binding domain (Fig 1*A*). However, a complete analysis of domains responsible for its subcellular localization has not yet been fully described. We began our study by generating three truncations, or fragments (F1, F2, and F3), of CP190 (Fig 1*A*). F1 (aa 1–209) contains the BTB domain and a region we refer to as the Linker (L) domain. F2 (aa 210–583) contains the previously identified centrosome and MT targeting domain [11], and a nuclear localization signal (NLS). F3 (aa 584–1096) encompasses the entire E-rich domain. To determine the subcellular localization of these fragments, we expressed GFP tagged versions of F1, F2, and F3 in *Drosophila* S2 cells and compared their distribution during mitosis to the full-length (FL) CP190-GFP (CP190^FL^) control. As expected, CP190^FL^ robustly localized to centrosomes (11/11 cells) and chromosomes (Fig 1*B*). To visualize the relatively weak spindle MT localization of CP190^FL^, it was essential to enhance the contrast of the image (Fig 1*B*, zoom, yellow arrowhead). This dim MT localization, seen in 10/11 mitotic cells, is not an artifact of image enhancement, as these cells were not stained for any spindle markers (such as MTs) that could bleed through from other fluorescent channels. Predictably, F3 did not localize to centrosomes (0/10 cells) or to spindle MTs (0/10 cells; Fig 1*B*). In contrast, F2 robustly localized to centrosomes (10/10 cells), but was not detectable on spindle MTs (0/10; Fig 1*B*). This was quite surprising since F2 contains the known MT binding domain. To investigate this further, we generated several truncations of F2, including a construct previously identified as the minimal centrosome and MT interacting region (F2^385-508^), which was sufficient for spindle localization (data not shown), suggesting that the spindle-targeting capabilities inherent to the F2^385-508^ construct were masked in the context of the larger F2 construct. Unexpectedly, F1 robustly localized to spindle MTs (18/21 cells, Fig 1*B*, yellow arrow), as well as centrosomes (21/21 cells). We therefore identified a novel region of CP190; sufficient for MT and centrosome-targeting that merited further investigation.

### Interphase S2 cells serve as a model to study CP190 MT association

**Fig 2:**
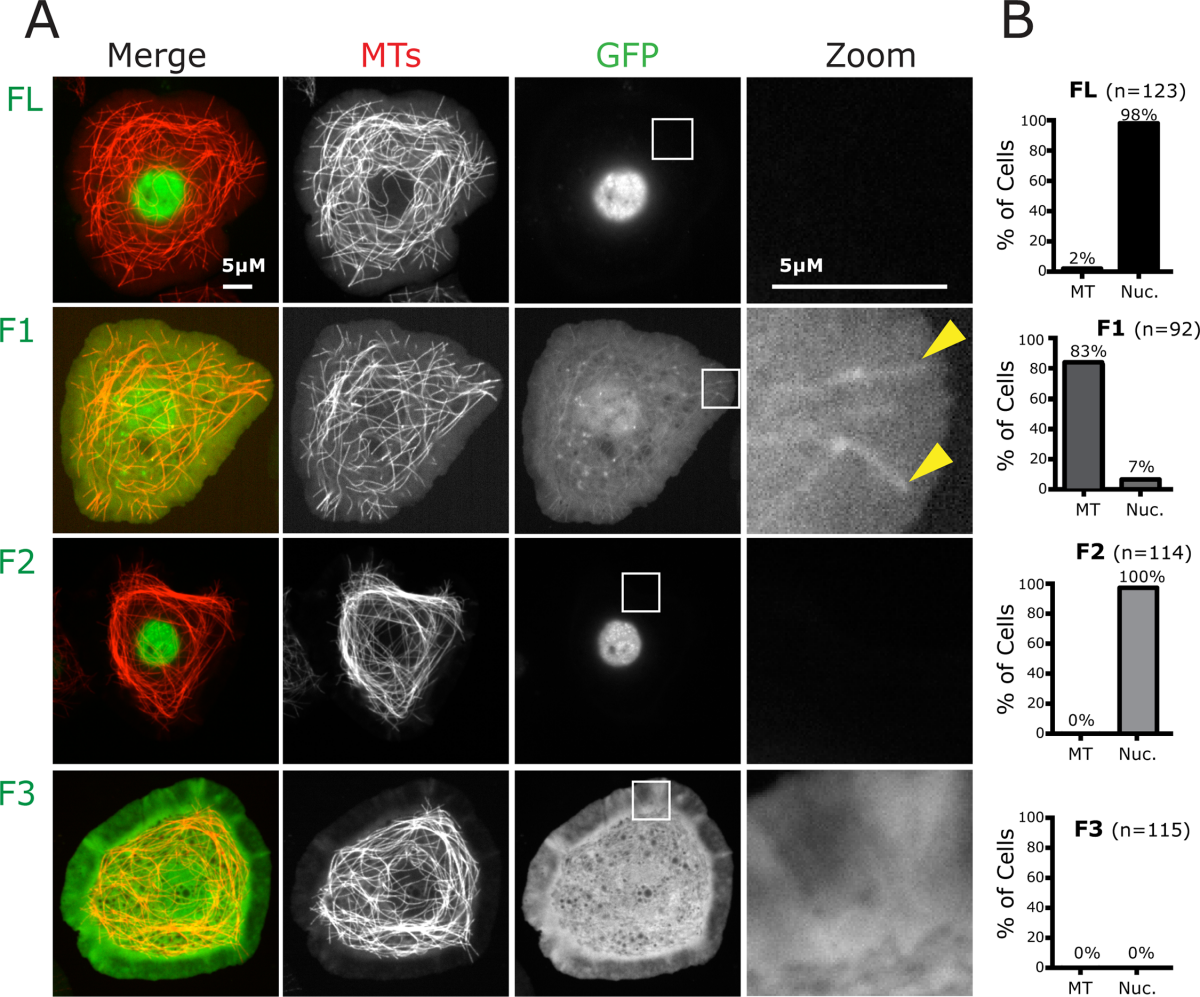
Interphase cells serve as an excellent model to study CP190 MT association. *A*, *Drosophila* S2 cells expressing GFP-CP190 constructs and TagRFP-Tubulin. Box in GFP channel is zoomed to highlight GFP localization to MTs (right column). F1 localizes to centrosomes and is unique in its localization to MTs during interphase, similar to what we document in mitosis (Fig 1*B*). *B*, Quantification of the percent of cells in which CP190 constructs co-localize with MTs or show nuclear (Nuc) localization. FL and F2 are localized to the nucleus during interphase. F3 is cytoplasmic during interphase. F1 localizes to MTs robustly during interphase (see S1 Fig). Scale bars = 5 μm.

While assessing the mitotic localization of F1, F2, and F3, we noticed that F1 also localized in a MT-like pattern in interphase cells (Fig 2*A*). In contrast, CP190^FL^ and F2 were predominantly found within interphase nuclei as expected (both contain the NLS), while F3 was diffusely cytoplasmic (Fig 2*A*). Additionally, even the minimal F2^385-508^ construct, which lacks the NLS but showed mitotic spindle localization, was unable to robustly interact with interphase MTs (data not shown). To assess the interphase localization of each fragment in more detail, we analyzed S2 cells co-expressing TagRFP-tubulin to label MTs and GFP fusions of CP190^FL^, F1, F2, or F3. Our quantification indicated that F1 was indeed associated with the MT network in over 80% of cells and robustly localized to centrosomes in 70% of cells (Fig 2*A* and 2*B*; S1 Fig). Notably, we did not observe any MT or centrosome localization of either F2 or F3 (Fig 2*A* and 2*B*; S1 Fig), suggesting that the recruitment of F2 to centrosomes during mitosis requires a mitotic-specific post-translational modification or additional factors that are not available during interphase. We also note that while F1 localized to MTs, there was also a diffuse, cytoplasmic pool. Whether mitotic-specific post-translational modifications or other factors would also promote F1-MT association outside of interphase remains to be determined.

Given the low mitotic index, the spatial restrictions of the mitotic spindle, and the poor quality of imaging rounded mitotic cells, we turned to interphase cells to further explore the F1-MT interaction as interphase S2 cells plated on concanavalin A adopt a flat morphology, highly amenable to imaging. In addition, we reasoned that endogenous CP190 found in the nucleus of interphase cells would be spatially segregated away from F1, allowing us to investigate the F1-MT association without the confounding complication of F1 potentially oligomerizing with endogenous CP190. Nevertheless, to directly address this potential complication, we confirmed that F1 is sufficient to associate with MTs in the absence of endogenous CP190 expression (S2 Fig; methods). Thus, interphase MTs are an ideal model for investigating the MT localization of F1.

### CP190 F1 is enriched on MTs

**Fig 3:**
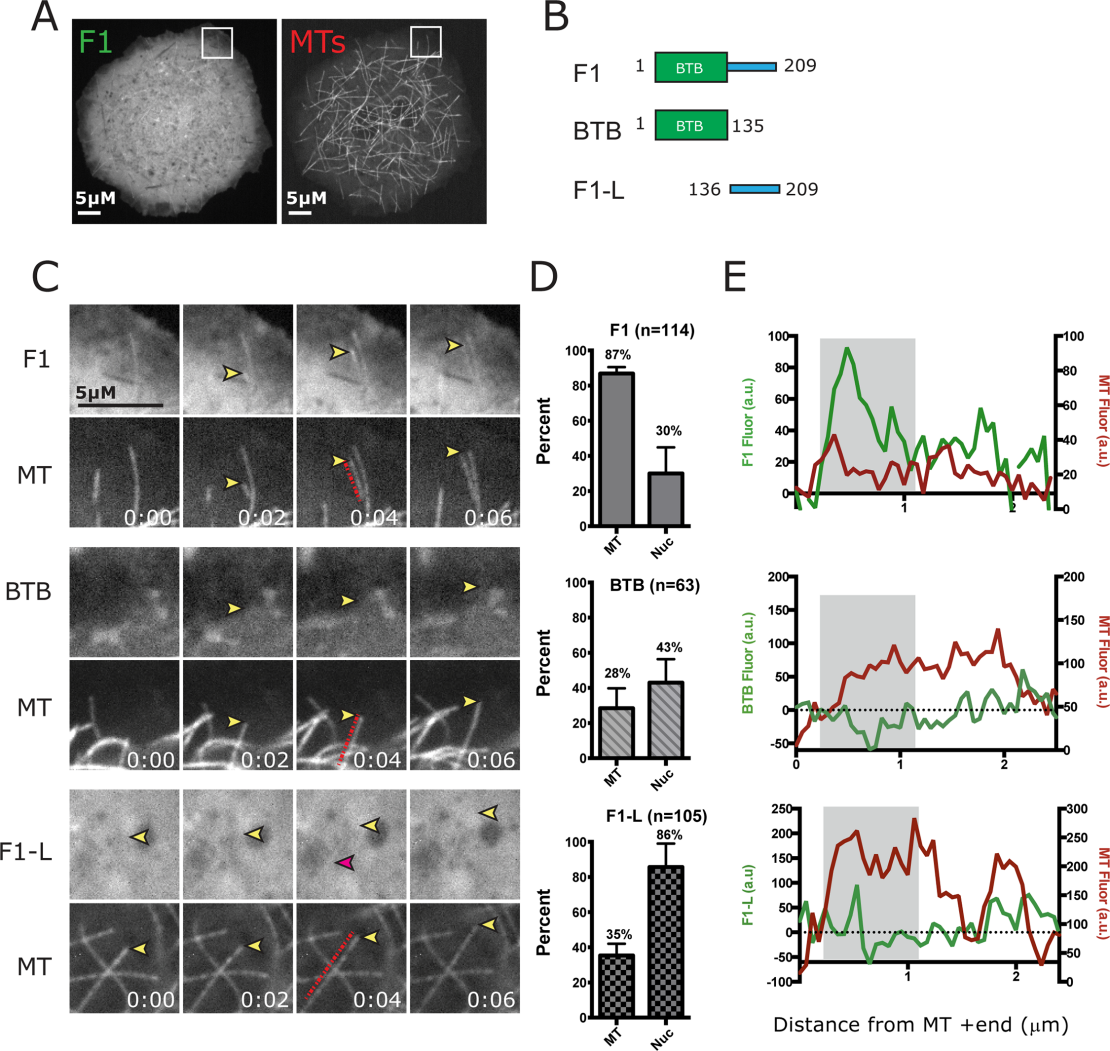
CP190-F1 is enriched at the plus ends of growing MTs. *A*, Images of S2 cells expressing GFP-F1 and TagRFP-Tubulin. Box indicates inset for zoom in C. *B*, Schematic of the CP190-F1 sub-fragments F1, BTB and Linker domain (F1-L) analyzed for MT association. *C*, Live-cell imaging of F1 reveals enrichment at the plus end of MTs. Yellow arrowhead indicates a growing MT plus end. *D*, Graphs indicate percent of cells with MT and Nuclear localization. *E*, Graphs shown to the far right are line scans along the MTs indicated in frames 0:04 (red dashed line) in C. X-axes are arbitrary fluorescence units (a.u.) and y-axis is distance in microns from the MT +tip end (coordinate x = 0). F1 linescan indicates its enrichment at the MT +ends (grey area on graph). In contrast, the BTB domain and F1-L show very weak localization to some MTs (pink arrowhead, grey box on graph). Scale bars = 5μm. Time = min:s.

Given the localization of F1 to the MT lattice, we sought to investigate the dynamics of F1 in live cells. We performed high-resolution time-lapse imaging of GFP-F1 and TagRFP-tubulin in cultured S2 cells (Fig 3*A*–3*C*). We found that in addition to decorating the entire MT length, F1 was particularly enriched at growing MT plus ends (Fig 3*C*). To more precisely define the MT targeting region within F1, we truncated F1 into two smaller fragments (Fig 3*B*): the BTB domain (BTB) and a linker region (F1-L). However, when analyzed in live cells, neither BTB nor F1-L robustly localized to MTs (Fig 3*C*–3*E*; middle and bottom panels). In a small percentage of cells (<25%), we note extremely weak MT association by the BTB domain and F1-L (Fig 3*C*, pink arrow). These results suggest that the BTB domain and the F1 linker collectively drive CP190 MT-localization.

### CP190 F1 directly binds MTs, but not EB1, *in vitro*

Based on our data, we hypothesized that F1 associates with MTs in one of two ways: 1) via interactions with the central MT plus end-binding protein, EB1 [32–34], or 2) via direct interaction with the MT lattice. To distinguish between these two possibilities, we tested the association of F1 with either EB1 or taxol-stabilized MTs *in vitro*. In many cases, proteins associate with the growing MT plus end via an EB1 binding SxIP motif (ser-x-ile-pro, surrounded by basic residues), which mediates direct binding to EB1 [33]. In addition, interaction of EB1 with centriole and centrosome proteins has been described previously [34]. We searched the F1 primary sequence and identified a putative SxIP-like EB1 binding motif (SGLP; aa 151–154) within F1-L (S3*A* Fig). Previous work has shown that mutagenesis of an SxIP motif to SNNN is sufficient to ablate EB1-dependent MT plus end association [34]. Therefore, to test if this motif in CP190 is required within F1 to mediate MT association, we mutated the SGLP sequence to SNNN in the context of F1 (F1^SNNN^; S3*A* Fig). Expression of GFP-F1^SNNN^ in S2 cells revealed that it has reduced localization to MTs (S3*B* Fig). While it remains formally possible that the F1^SNNN^ mutation is detrimental to overall protein structure, these results are most consistent with a mechanism whereby F1-L directly binds EB1 via the SGLP motif.

To test whether F1 directly interacts with EB1, we assayed for an EB1-F1 interaction *in vitro* with purified components. Using size exclusion chromotography, we detected no significant peak shift of F1+EB1 compared with either protein alone (S3*C* Fig). We conclude that F1 does not robustly bind EB1, at least not under the stringent conditions of our *in vitro* assay. It is possible that the F1 interaction with EB1 is transient. Alternatively, the SGLP motif in the linker may be involved in a direct MT interaction independent of EB1.

**Fig 4:**
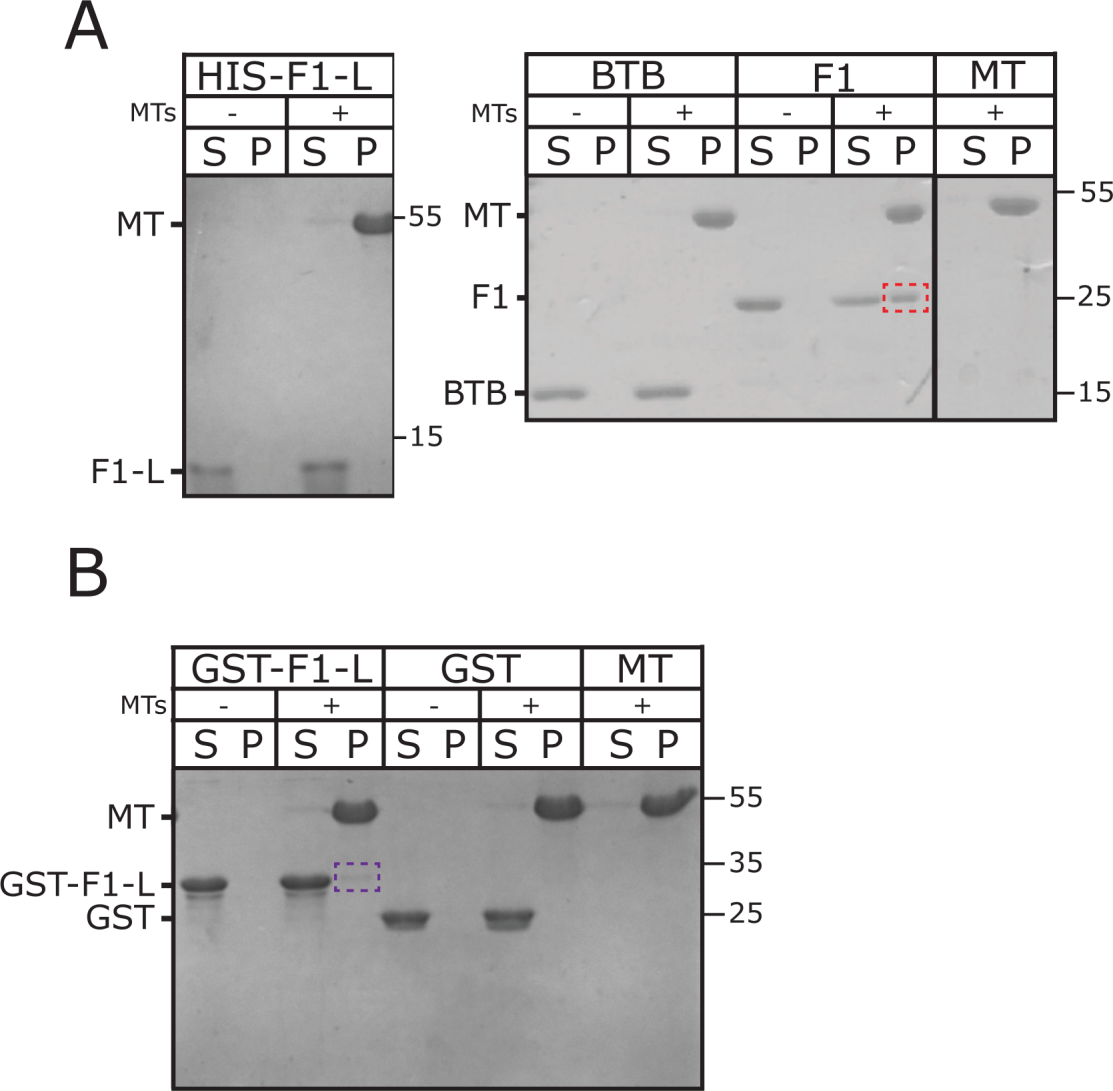
CP190-F1 binds directly to MTs. MT co-sedimentation assay with purified components. *A*, Coomassie stained gel shows CP190-Linker (F1-L), CP190-BTB domain and CP190-F1 fragment incubated without (-) and with (+) MTs followed by high-speed centrifugation. Supernatant (S) and pellet (P) fractions are run separately. F1 co-sediments with MTs (red dotted box), while neither the F1-L linker nor the BTB domain show MT-binding. *B*, Coomassie stained gel shows the F1-L linker artificially dimerized as a GST fusion (GST-F1-L) and GST alone incubated without (-) and with (+) MTs followed by high-speed centrifugation. Supernatant (S) and pellet (P) fractions are run separately. Although we were not able to fully recapitulate F1 MT binding, dimerized linker (GST-F1-L) is able to weakly associate with MTs (purple dotted box).

To test the second hypothesis that F1 interacts directly with MTs, we performed a MT co-sedimentation assay using purified, taxol-stabilized MTs and purified F1 (Fig 4*A*). In the absence of MTs, F1 was found exclusively in the supernatant fraction. Upon the addition of taxol-stabilized MTs, significant amounts of F1 appeared in the pellet fraction, indicating a direct interaction between F1 and MTs *in vitro* (Fig 4*A*). To further narrow the interaction domain, we generated purified protein of the two F1 fragments, the BTB domain and F1-L. Consistent with our cellular findings, neither the linker, nor the BTB domain alone is sufficient for MT association (Fig 4). Therefore a combination of F1-L and BTB activity is required for MT binding.

### The CP190 BTB domain is highly conserved across species

**Fig 5:**
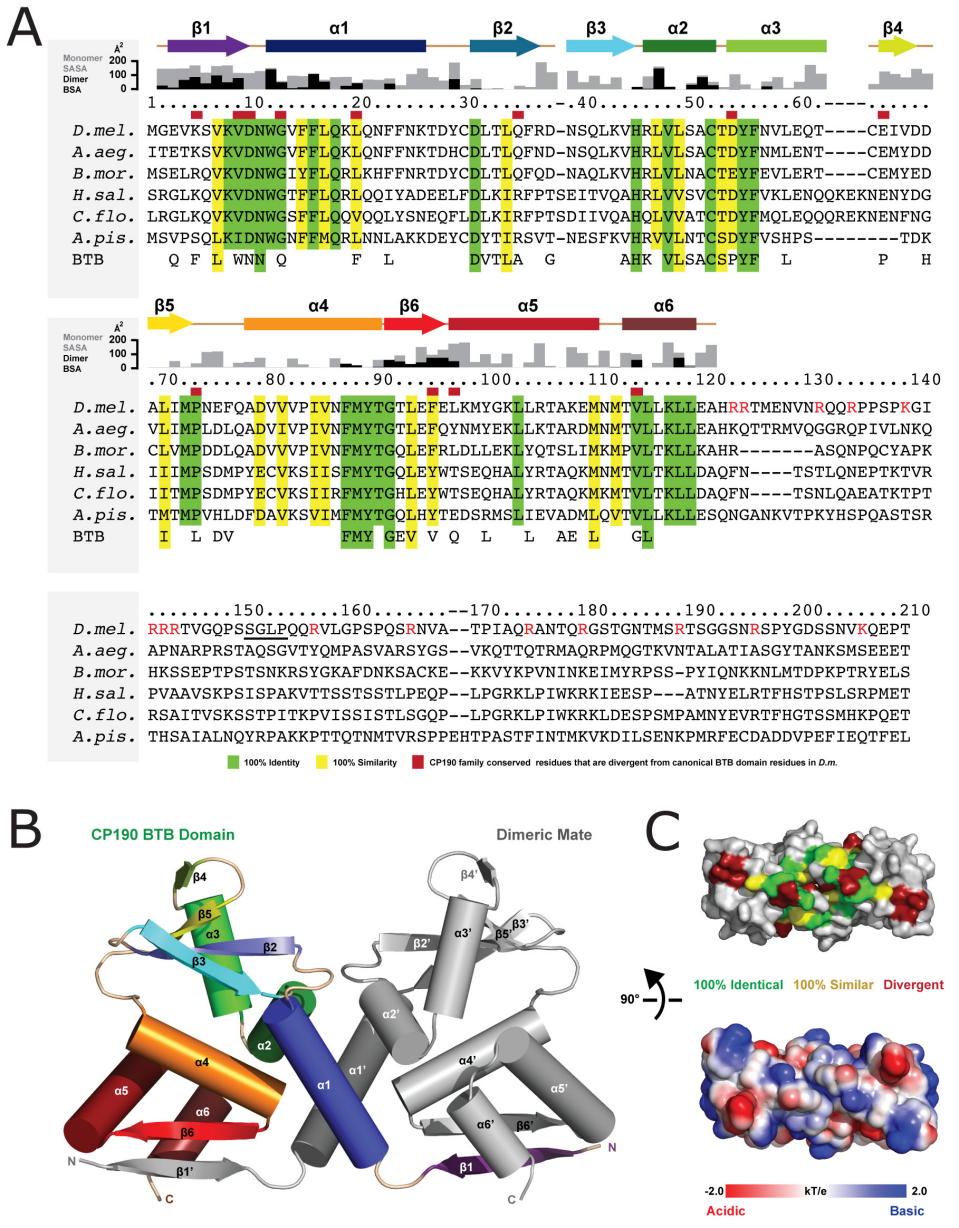
The CP190 BTB domain is highly conserved across species. CP190 F1 region sequence alignment across six species shows a high level of conservation within the CP190 BTB domain. Residue numbers correspond to *Drosophila melanogaster* CP190. Conservation is mapped on the alignment. Residues with 100% identity are mapped in green and residues with 100% similarity are mapped in yellow. Below the CP190 sequences (last row) are displayed residues that are highly conserved among other *Drosophila melanogaster* BTB domain containing proteins (not CP190) and are likely involved in the BTB domain fold. Residues from this set that are divergent in CP190 are indicated above the *Drosophila* CP190 sequence by a red rectangle. This alignment suggests that there are residues important for the BTB domain structural fold and others that are specifically unique to CP190. Secondary structure is mapped on the alignment. Arrows indicate β-sheets and rectangles indicate α-helices. Red letters in the linker region highlight basic (R and K) residues. The SGLP motif is underlined. Solvent accessible surface area (SASA) of a theoretical monomer as well as the buried surface area (BSA) of the homodimer is plotted above the alignment. *B*, The BTB domain adopts a dimeric fold and makes extensive structural contacts with its dimeric mate. One BTB domain is shown in color with secondary structure elements colored as in Figure 5*A*. The dimeric mate is colored grey. The β1 and β6’ strands from dimeric mates form an antiparallel, two-stranded β-sheet. *C*, The BTB domain shown in surface representation, rotated 90° about the x-axis relative to the orientation shown in B. Conservation is colored on the structure (above) following the scheme in A. Electrostatics are indicated on the structure below.

To map conservation in the F1 region, we aligned *Drosophila* F1 with the corresponding N-terminal region of CP190 homologs from five other diverse insects (Fig 5*A*). The N-terminal 120 amino acids that constitute the BTB domain show the highest degree of conservation. Many of the BTB domain residues conserved across CP190 members are also conserved across other *Drosophila* BTB-domain containing proteins and are involved in BTB domain structure (Fig 5*A*, lower line in the alignment). However, we note that CP190 also contains conserved residues that have diverged from the position-equivalent conserved residues found in other *Drosophila* BTB domains (Fig 5*A*, red boxes), suggesting that the CP190 BTB domain may have evolved a specific function, unique from other BTB domain proteins. In contrast to the conserved nature of the CP190 BTB domain, we find that the F1 C-terminal linker region is poorly conserved across species, but does have a high percentage of basic residues often found in MT-binding proteins (Fig 5*A*).

### Structure of the CP190 BTB domain reveals a conserved fold and dimerization mode

**Fig 6:**
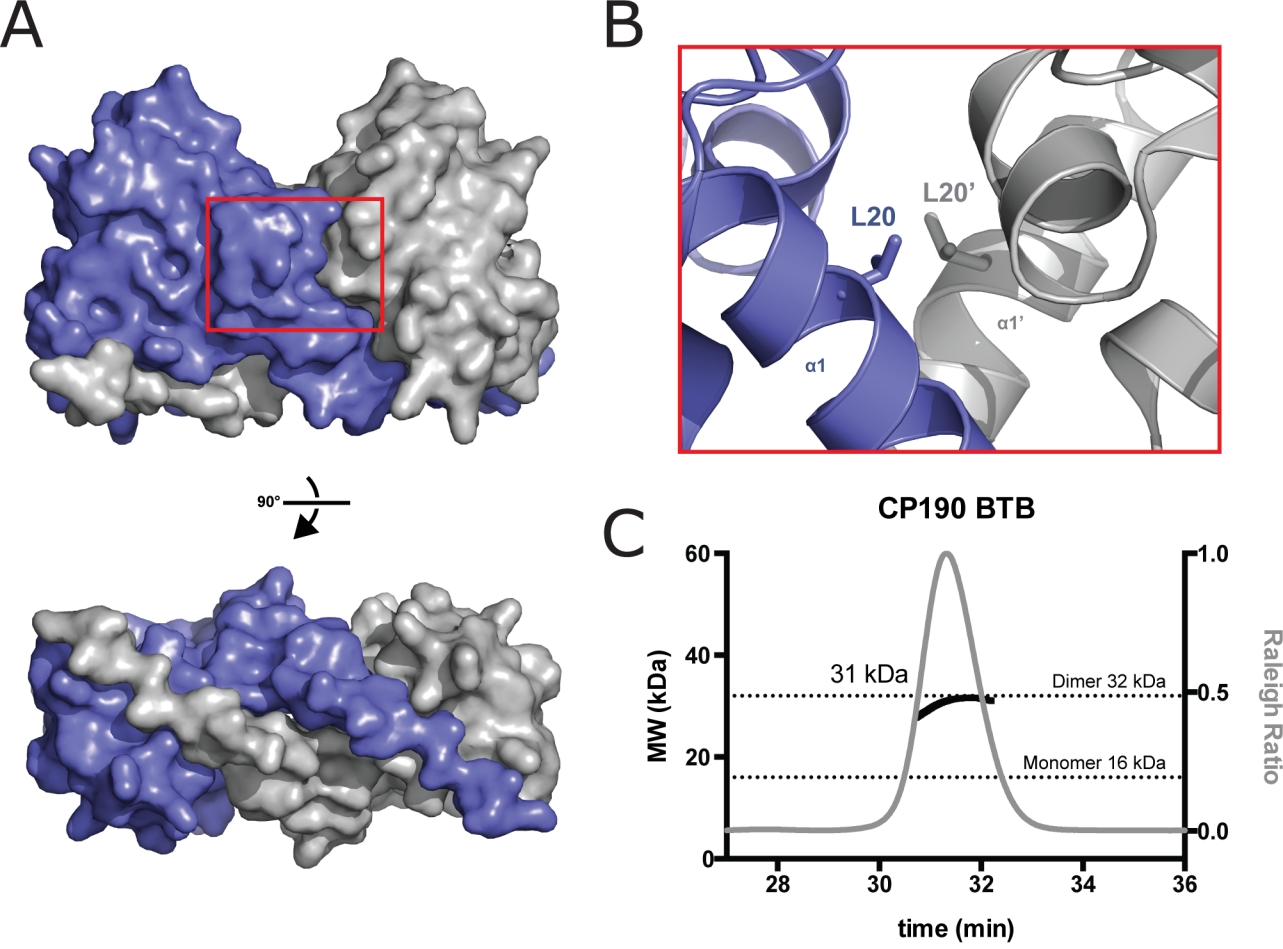
The BTB domain exists as a dimer and is critical for F1 MT binding. *A*, Space-filling model showing homodimeric mates in the crystal lattice with one molecule colored purple, the other grey. The β1 strands wrap along the length of the opposing molecule. *B*, Zoom of the dimer interface shows the hydrophobic leucine (L20) residue that was mutated to a charged glutamic acid residue (L20E). This mutation destabilizes the BTB domain at the dimer interface. *C*, CP190’s BTB domain exists as a stable dimer in solution as shown by SEC-MALS. The x-axis is the time of the run in minutes, the left y-axis is the MW (black: kDa), and the right y-axis is the Raleigh ratio (grey). The predicted CP190 BTB domain monomer and dimer molecular weights are indicated by dotted lines (16 and 32 kDa respectively). The experimentally determined mass of the eluted CP190 BTB domain is plotted as a black line and corresponds to a homodimer.

To gain structural insight into the conserved CP190 BTB domain, we purified and crystallized native and selenometionine-substituted BTB (residues 1–135). We collected native and selenium peak SAD data sets on single crystals to 2.5 and 2.7 Å resolution, respectively. Crystals belong to the space group P3_2_21 and contain one CP190 BTB molecule in the asymmetric unit. The structure was built and refined to 2.5 Å resolution, yielding R and R_free_ values of 21.9 and 25.9, respectively. The final model contains residues 2–121. The BTB domain forms a homodimer across a crystallographic 2-fold axis, adopting a canonical homodimeric structure as found in the BTB-ZF subfamily of BTB domains [17,35]. The BTB homodimer has dimensions of ~60 x 35 x 25 Å (Fig 5*B*). Each chain in the homodimer has an α-helical core composed of six helices, α1- α6, flanked by a four stranded β-sheet (β3-β2-β5-β4) and a two-stranded inter-molecular β-sheet formed by β6 and β1' from the homodimeric mate. Residues that are conserved across CP190 family members, as well as those residues that are conserved across *Drosophila* BTB domains but are divergent in CP190, cluster to a basic face of the homodimer where the N- and C-termini reside (Fig 5*C*). In the dimeric structure, β1 extends along the base of its dimeric mate forming extensive anti-parallel β-sheet hydrogen bonding augmented through extensive van der Waals contacts mediated by conserved hydrophobic side chains in β1. The central region of the BTB domain is comprised of six α-helices. On either side of the core, a pair of β-sheets stabilizes the BTB domain fold. Hydrophobic residues are buried at the dimer interphase. Key dimer contacts are made at this hydrophobic interface as well as a reciprocal N-terminal β-strand exchange in which β1 extends along the base of the homodimeric mate, forming a two-stranded antiparallel β-sheet with β6'. Each BTB domain buries 1700 Å^2^ at the dimer interface (Fig 6*A*). Prime dimerization contacts are mediated by β1, α1, α2, β6', and α6 and involve both hydrogen bonding as well as van der Waals contacts (Fig 5*B*). Along the homodimer’s two-fold axis, α1 makes key hydrophobic interactions with α1' from the homodimeric mate. Using the Dali server, we found that the CP190 BTB domain is most similar to the BTB domain from PLZF [35] (PDB 1BUO), with an rmsd of 1.1 Å. The PLZF BTB domain also belongs to the BTB-ZF subfamily, is an obligate homodimer, and likewise uses its key β1 and α1 structural elements to mediate symmetric dimerization.

### CP190 BTB is a homodimer in solution

At the core of the CP190 BTB homodimer is an α1-α1' interface where a key conserved residue, L20 (Fig 5*A*), packs against its homodimeric mate, L20' (Fig 6*B*). To test whether our crystallographic dimer interface is a *bona fide* dimerization interface, we first analyzed whether the BTB domain was a dimer in solution. We analyzed the oligomeric state of the BTB domain using SEC-MALS. BTB eluted from the size exclusion column as a single Gaussian peak with an experimentally determined molecular weight of 31 kDa, corresponding to a homodimer (monomer molecular weight: 16 kDa, homodimer molecular weight 32 kDa) indicating that the CP190 BTB domain forms a stable homodimer in solution (Fig 6*C*). We next tested whether mutating L20 to glutamate would compromise domain stability and/or the homodimeric state by introducing repulsive charges at the dimer interface. Generating this mutation in the context of the BTB domain alone (BTB^L20E^) rendered the protein insoluble when expressed in *E*. *coli* (S4 Fig), likely by destabilizing the dimer interface and exposing hydrophobic residues. We conclude the highly conserved L20 residue is critical to support homodimerization.

### Dimerization of CP190-F1 is important for MT association

**Fig 7:**
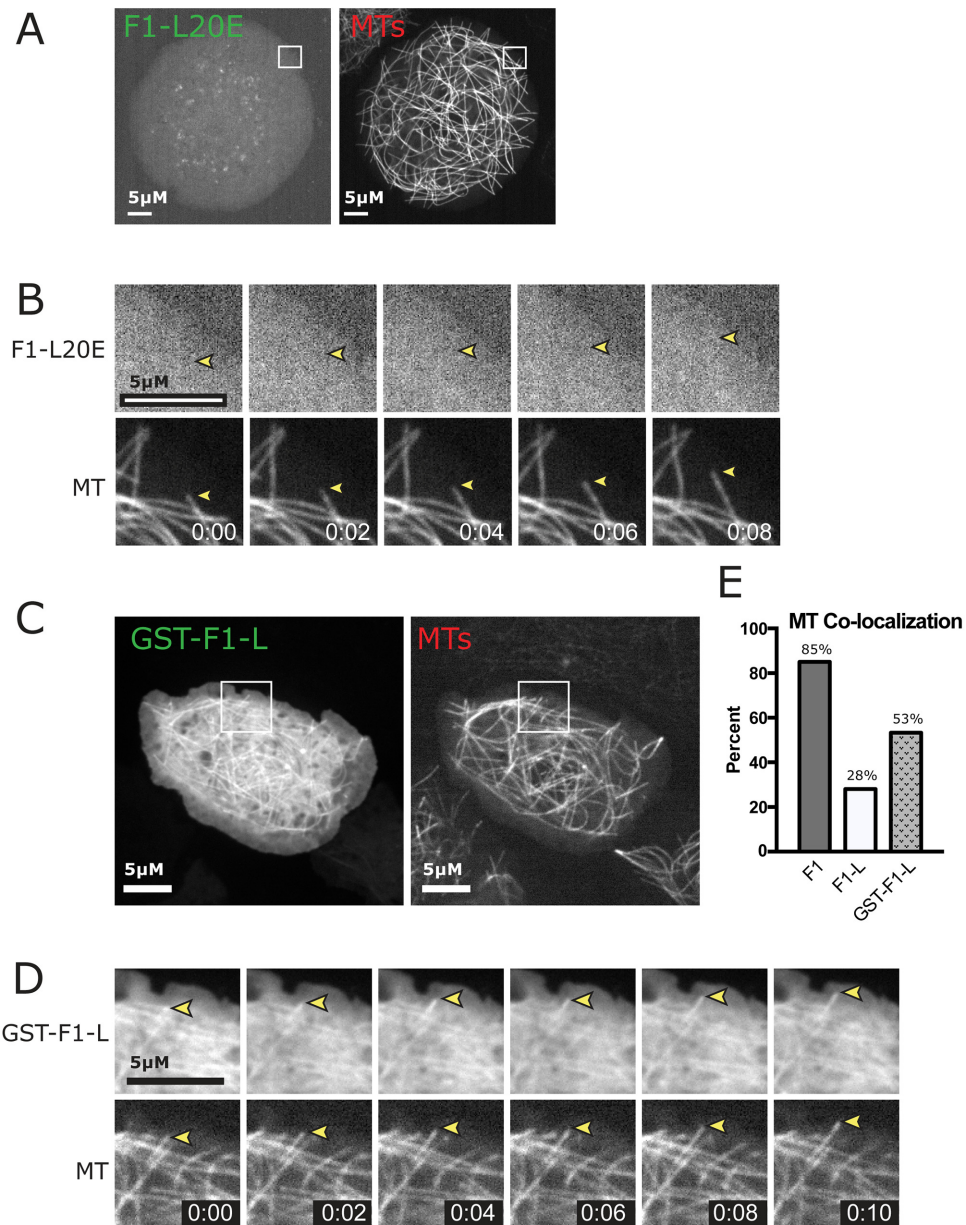
Artificially dimerized F1-L localizes to MT. *A*, S2 cell expressing the F1-L20E dimerization interface mutant and TagRFP-Tubulin. F1-L20E is unable to localize to MTs *in vivo*. White box indicates zoom for B. *B*, Time-lapse from boxed region showing that F1-L20E does not localize to MTs (yellow arrowhead). *C*, S2 cells expressing GST-F1-L and TagRFP-Tubulin. White box indicates zoom for D. *D*, Time-lapse images from boxed region in C showing robust localization of GST-F1-L to the MT lattice. Scale bars = 5 μm. *E*, Percent of cells showing MT-Colocalization of F1, F1-L and GST-F1-L. Localization is partially rescued with artificially dimerized F1-L. Time is indicated in min:s.

To test whether BTB-mediated dimerization is important for MT association, we introduced the L20E dimer interface mutation into F1 (F1^L20E^) and expressed it in *Drosophila* S2 cells along with TagRFP-tubulin. Our live imaging revealed that F1^L20E^ is primarily cytoplasmic and does not associate with MTs (Fig 7*A* and 7*B*). This result strongly suggests that dimerization of F1 through the BTB domain is important for F1 to localize to MTs. To directly test whether dimerization promotes F1-L MT association, we artificially dimerized F1-L by replacing the BTB domain with GST, generating a GST-F1-L fusion and introducing it into S2 cells. Our live cell analysis shows a striking rescue of MT localization (Fig 7*C* and 7*D*; compare with F1 in Fig 3*C*). The importance of dimer formation was confirmed using a second dimerization method in which the coiled coil homodimerization domain from *S*. *cerevisiae* GCN4 was fused to F1-L. This method also resulted in the rescue of F1-L MT localization (S5 Fig). To further investigate the ability of dimerized linker to associate with MTs we performed a MT co-sedimentation assay with purified GST-linker (GST-F1-L). While F1-L alone is unable to associate with MTs, upon dimerization with GST, GST-F1-L is able to weakly associate with MTs *in vitro* (Fig 4B). These data confirm that the linker domain is able to confer MT association, albeit at reduced levels as compared to full-length F1. Therefore, the BTB domain likely contains additional determinants, beyond dimerization alone, which are important for association with MTs. Alternatively the mode of BTB dimerization (compared with GST- or GCN4-mediated dimerization) could afford unique structural constraints for linker-MT association. These two independent lines of evidence support a mechanism where dimerization of the CP190 linker region is crucial for MT lattice localization.

### CP190-Linker region is important for MT spindle organization in brain stem cells

Despite the localization of CP190 to centrosomes and its ability to bind MTs, it was unclear what cytoskeletal role, if any, CP190 plays *in vivo*. Having characterized this new MT-binding region, we sought to determine its possible importance for MT and centrosome function in cells. To this end, we specifically deleted the F1 linker region from the endogenous *cp190* gene using CRISPR/Cas9 technology in *Drosophila melanogaster* to produce a new allele we term *cp190* ^*ΔL*^ (S6*A* Fig). PCR and sequence analysis confirmed that we had properly deleted the linker and preserved the proper reading frame (S6*B* Fig). Of note is that we generated three independently verified CRISPR lines (*cp190* ^*ΔL*^,*cp190* ^*ΔL-1*^, *cp190* ^*ΔL-2*^). We report data only on the *cp190* ^*ΔL*^ line, but the other independent lines show nearly identical results. Western blot of *cp190* ^*ΔL*^ shows that CP190 ^ΔL^ protein is present and stable, unlike a previously described hypomorphic CP190 allele (*cp190* ^*H4-1*^) that produced no detectable protein (S6*C* Fig). To avoid the complication of possible CRISPR off-targets, all experiments were performed using trans-heterozygote animals carrying *cp190* ^*ΔL*^ and a Deficiency (*Df* ^*p11*^) that removes the *cp190* locus. *cp190* ^*ΔL*^/ *Df* ^*p11*^animals are fully viable, allowing for detailed analysis of centrosomes and mitotic spindles.

**Fig 8:**
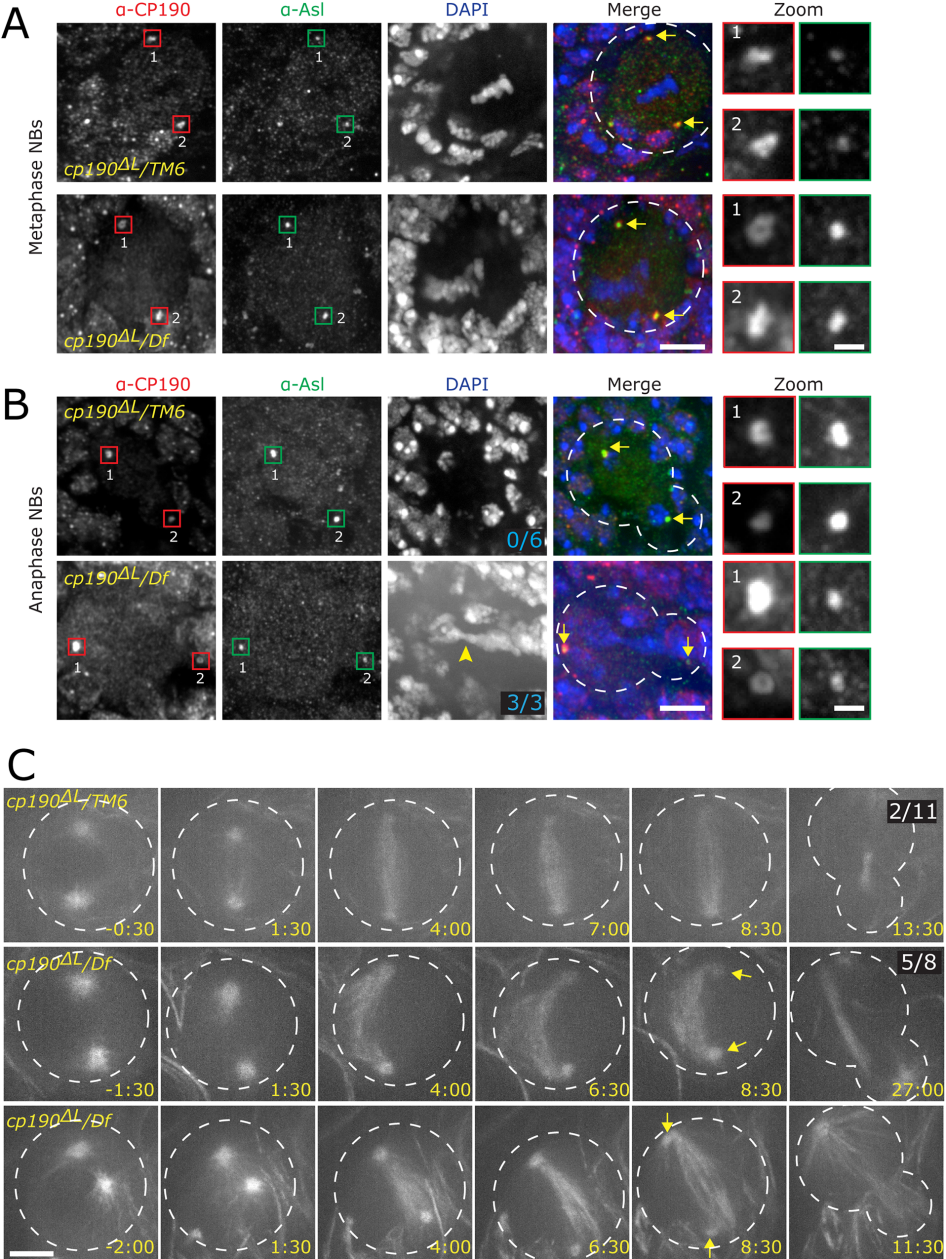
CP190-L is important for spindle formation in developing brain. *Drosophila* NBs were fixed and stained as indicated. *A*. Metaphase NBs are shown with CP190 in red, Asl to mark the centrosome in green, and DAPI in blue. WT control (*cp190* ^*ΔL*^ */TM6*) is in the top row and mutant *cp190* ^*ΔL*^ */ Df* ^*p11*^ in the bottom row. Boxed regions indicating centrosomes are magnified and shown on the far right panels (numbers next to centrosome indicate which zoomed centrosome is displayed). Yellow arrows point out centrosomes in the merged channel. White dotted line indicates NB outline. *B*.Fixed anaphase NBs. Labeling is the same as in *A*. Note a lagging chromosome in *cp190* ^*ΔL*^ */ Df* ^*p11*^ (yellow arrowhead), which is never seen in WT (frequency indicated in DAPI channel). Scale bar for *A* and *B* = 5μm, zoom = 1μm. *C*, Live imaging of MTs in WT (*cp190* ^*ΔL*^ */TM6*) and *cp190* ^*ΔL*^ */ Df* ^*p11*^mutant NBs, note bent spindle (frequency indicated in right most image) and detached centrosome (yellow arrows) in mutant cell. Scale bar = 5μm. Panels in C from top to bottom correspond to S1 Movie, S2 Movie and S3 Movie, respectively.

Previous work on the *cp190* ^*ΔM*^ allele (which deleted the previously characterized MT binding region) showed that CP190^ΔM^ protein does not localize to centrosomes, but from the image presented in their manuscript, it appears to localize weakly to the spindle [22], presumably through the linker domain MT binding site we have identified. The authors also report that *cp190* ^*ΔM*^ homozygous flies only survive for a few days as adults, but attribute this lethality to transgene overexpression and not an essential centrosome or MTs role for CP190. Furthermore, this study did not note any centrosome or MT defects. To investigate the role of the MT-binding domain in the linker, we analyzed *cp190* ^*ΔL*^/*Df* ^*p11*^ *Drosophila* neural stem cells (neuroblasts, NBs). We fixed and stained *cp190* ^*ΔL*^/ *Df* ^*p11*^NBs using a polyclonal antibody made to CP190 lacking the BTB domain [14] and show that CP190^ΔL^ protein localizes to the nucleus in interphase and to centrosomes in mitosis, similar to wild type CP190 (Fig 8*A*). Most importantly *cp190* ^*ΔL*^ mutant NBs show DNA condensation defects, where the chromosomes consistently occupy a greater area. In addition, these condensed chromosomes are frequently not centered within the cell (Fig 8*A*, *DAPI*), suggesting that the mitotic spindle is defective in some way.

To investigate spindle formation and maintenance in *cp190* ^*ΔL*^ mutant NBs, we introduced GFP-Tubulin into the *cp190* ^*ΔL*^/ *Df* ^*p11*^ background. Live imaging confirmed our suspicion and revealed abnormally curved spindle in 62% (n = 5/8, S2 Movie) of cells we examine in mutant flies, a phenotype rarely (18%, n = 2/11; S1 Movie) seen in controls (Fig 8*C*). Another surprising phenotype was centrosome detachment from the spindle (Fig 8*C*; S3 Movie) following spindle formation. These results support a cytoskeletal role for CP190 that requires interaction with MTs via the linker domain.

## Discussion

### CP190 uses multivalent determinants to localize to the spindle during mitosis

CP190 is a large multi-domain protein that has a distinct localization pattern that is tightly coordinated with the cell cycle. During interphase it is restricted to the nucleus where it serves as a key component of chromatin insulator complexes that spatially organize the genome [14]. Upon nuclear envelope breakdown, a population of CP190 redistributes to the centrosomes and the spindle. Extensive domain analyses and structure-function work has been completed on CP190 in *Drosophila* syncytial embryos to examine its interphase role as a chromatin insulator, while its role during mitosis remains unclear. Importantly, studies subsequent to the initial CP190 domain mapping [22,23] drew upon conclusions reached using constructs that lacked much of the N-terminal region [11,36]. Our study here focusses on this region, which includes the BTB domain (aa 1–135) and the linker (F1-L aa 136–209).

We have identified a novel centrosome targeting and MT interaction domain within the N-terminus of CP190 and our analysis of CP190-MT interactions unveiled a role for CP190 in organizing MTs during mitosis. While the F1-L linker region displays weak MT localization activity, BTB domain-mediated dimerization robustly enhances the linker’s MT binding, likely through avidity, as replacing the BTB domain with a non-native dimerization domain (GST or GCN4) also enhances linker-MT targeting. Our structure of the CP190 BTB domain reveals a homodimeric fold that is similar to the PLZF BTB domain structure. SEC-MALs studies confirm the homodimeric nature of the CP190 BTB domain. Further, mutagenesis of a key residue at the dimer interface (L20) yielded insoluble protein, suggesting that the dimer interface observed in the crystal structure is *bona fide* and integral to protein structure. The bottom face of the CP190 BTB homodimer where the β1 strand and the N- and C-termini lie (Fig 5*C*) shows the highest relative degree of conservation/unique determinants suggesting that this face of the BTB homodimer may play a functional role in insulator complex association. While preparing this manuscript, a similar structure of the CP190 BTB domain was published that aligns well to our structure, serving as independent confirmation of the structure [17].

### Mitotic functions of CP190-linker

CP190’s complex, multi-domain features allow for dynamic localization to several key mitotic elements (centrosome, MTs and DNA). Despite this, no mitotic functions of CP190 have been ascribed to date. Prior studies had probed the role of CP190 in mitosis by examining CP190 constructs that lacked the previously identified MT and centrosome-targeting domain [11,23]. The major conclusion from those studies was that the CP190 MT and centrosome targeting domain was not essential, and thus CP190’s function was solely as a chromatin insulator and that although it localized to centrosomes and MTs during mitosis, it did not play functional role in mitosis. Here, we sought to further investigate the mitotic roles of CP190 by analyzing the linker region which we have shown directly interacts with MTs. Deleting the CP190-Linker at the endogenous locus by CRISPR afforded us the ability examine the function of the CP190-Linker within a live organism without the complications of over- or under-expression. Although *cp190* ^*ΔL*^ flies are viable and CP190^ΔL^ protein can still localize to centrosomes, our fixed and live cell analysis has revealed CP190-Linker-specific mitotic spindle defects. Specifically, deletion of the CP190 linker region leads to curved spindles that likely lack proper force balance between mitotic elements. Furthermore, the centrosomes appear to detach from the spindle poles. This suggests that CP190 could play a role in augmenting centrosomes:spindle MT attachment. It is important to highlight that *cp190* ^*ΔL*^ mutant flies are viable as a permanent stock can be maintained. Given that loss of CP190 insulator function causes lethality [23], it suggests that insulator function is maintained in *cp190* ^*ΔL*^ flies. It further suggests that the MT and centrosome phenotypes documented in *cp190* ^*ΔL*^ are a result of perturbing a direct cytoskeletal role of CP190, and not an indirect role of altering another spindle component at the transcriptional level via its insulator function.

In summary, we have used *in vivo* imaging, *in vitro* biochemical analysis and crystallography to identify and characterize a novel CP190 MT binding domain. Moreover, we use gene editing to test the role of this domain in live animals to uncover a cytoskeletal role for CP190. Future work will address how CP190 precisely influences MTs to ensure proper spindle function and centrosome attachment.

## Supporting Information

S1 Fig CP190-F1 localizes to centrosomes in interphase. S2 cells transfected with CP190 constructs (green) were fixed and stained for the centrosome marker Asterless (Asl, red) and scored for centrosome localization. White arrow indicates the centrosome in the zoomed inset. Scale bar = 10 μm.
(TIF)

S2 Fig CP190-F1 localizes to MTs in the absence of endogenous CP190. *A*. S2 cells were treated with dsRNA to knockdown endogenous CP190 and transfected with CP190 F1. Western blot shows effective CP190 knockdown (KD). A loading control (tubulin) is shown below. MW is indicated at left (kDa). *B*. Quantification of MT co-localization was completed in control and knockdown backgrounds using live cell imaging. The observed frequency of cells displaying CP190 F1 MT co-localization is on par with data presented in Fig 2B. We note, however, that the observed frequency of cells displaying CP190 F1 nuclear localization was significantly higher when analyzed using live cell imaging rather than fixed cell imaging. This difference is likely due to the harsh fixation protocol used.
(TIF)

S3 Fig The CP190 F1-L linker region does not stably interact with EB1 *over gel filtration*. *A*, Analysis of CP190 F1 revealed an SxIP–like motif in the linker (F1-L) region (yellow). A F1 construct in which the SxIP-like motif residues were mutated to SNNN was analyzed using live-cell imaging. *B*, Cells were transfected with F1^SNNN^ and TagRFP-Tubulin. Localization of CP190 GFP-F1^SNNN^ to the nucleus, MTs, or diffuse localization the cytoplasm, was quantified. There is a significant reduction of CP190 GFP-F1^SNNN^ as compared to WT F1 (Fig 2). *C*, Sizing column elution profile of EB1 (green), F1 (red), and EB1+F1 (blue). No significant peak shift is observed indicating that EB1 does not interact robustly with F1.
(TIF)

S4 Fig BTB dimerization is important for protein stability. Mutating the BTB domain at the dimer interface renders the protein insoluble. Coomassie gels showing whole cell lysates from transformed *E*. *coli* growing the indicated protein: Uninduced (Uni.), Induced (In). Both BTB-WT and BTB L20E are produced in *E*. *coli*. Red boxes highlight insoluble protein in the pellet (P), and soluble protein in the supernatant (S) after lysis and centrifugation. BTB-WT has a soluble fraction, whereas BTB-L20E does not.
(TIF)

S5 Fig BTB dimerization is important for localization of CP190 to MTs. S2 cells transfected with The GCN4 dimerization domain fused to F1-L rescues MT localization activity, indicating that dimerization is important for localization to the MT lattice. Bar = 10 μm.
(TIF)

S6 Fig Generation of *cp190* ^*ΔL*^ allele. *A*. CP190 genomic locus. 5’ and 3’ UTR are in grey, exons are numbered in blue boxes, black lines indicated introns, red box in intron 2 is the linker region, PAM guide RNA site is indicated by black arrow. Below the genomic locus is a schematic of the repair construct. Primers used to screen for linker deletion in CRISPR flies are indicated in orange. Line above indicated sequence regions. *B*. PCR of control yw fly and a heterozygous *cp190* ^*ΔL*^ fly. Expected sized are indicated to the right. *C*. Western blot shows that protein is produced in the *cp190* ^*ΔL*^ fly.
(TIF)

S1 Movie Time-lapse sequence showing spindle assembly and mitotic exit in control (*cp190* ^*ΔL*^ */TM6)* NBs. Time is in min:s and is relative to nuclear envelope breakdown.
(AVI)

S2 Movie Time-lapse sequence showing spindle assembly and mitotic exit in mutant (*cp190* ^*ΔL*^ */Df* ^*P1*^) NBs. Note curved spindle and detachment of centrosomes. Time is in min:s and is relative to nuclear envelope breakdown.
(AVI)

S3 Movie Time-lapse sequence showing spindle assembly and mitotic exit in mutant (*cp190* ^*ΔL*^ */Df* ^*P1*^) NBs. Note slight curved spindle and detachment of centrosomes. Time is in min:s and is relative to nuclear envelope breakdown.
(AVI)
