# Supplementary figures and images for: Newly Characterized Region of CP190 Associates with Microtubules and Mediates Proper Spindle Morphology in Drosophila Stem Cells
Source: PLoS One. 2015 Dec 9;10(12):e0144174. doi: 10.1371/journal.pone.0144174 (PMC4674064; doi:10.1371/journal.pone.0144174)

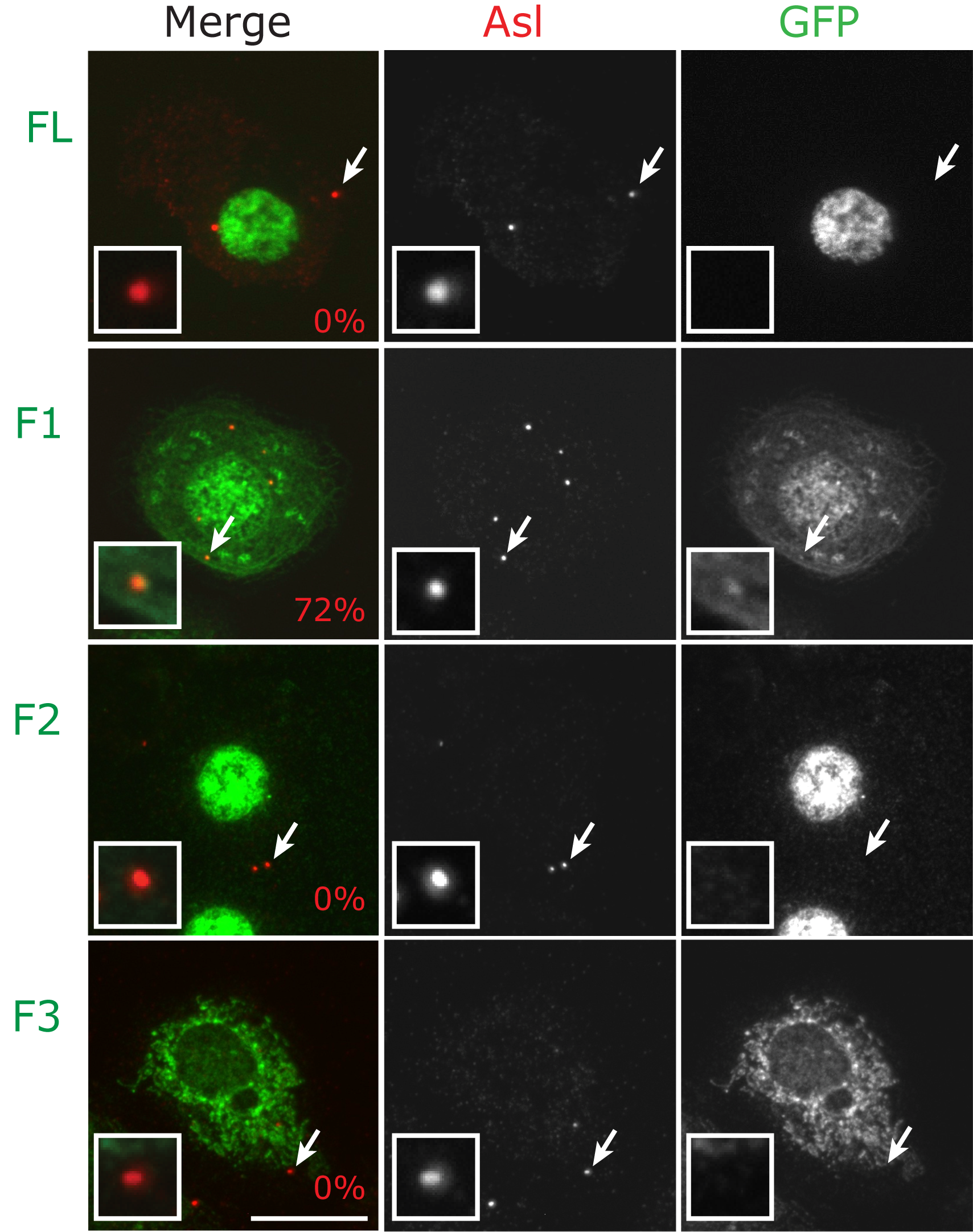

Supplement: S1 Fig — S2 cells transfected with CP190 constructs (green) were fixed and stained for the centrosome marker Asterless (Asl, red) and scored for centrosome localization. White arrow indicates the centrosome in the zoomed inset. Scale bar = 10 μm. (TIF) [file pone.0144174.s001.tif]

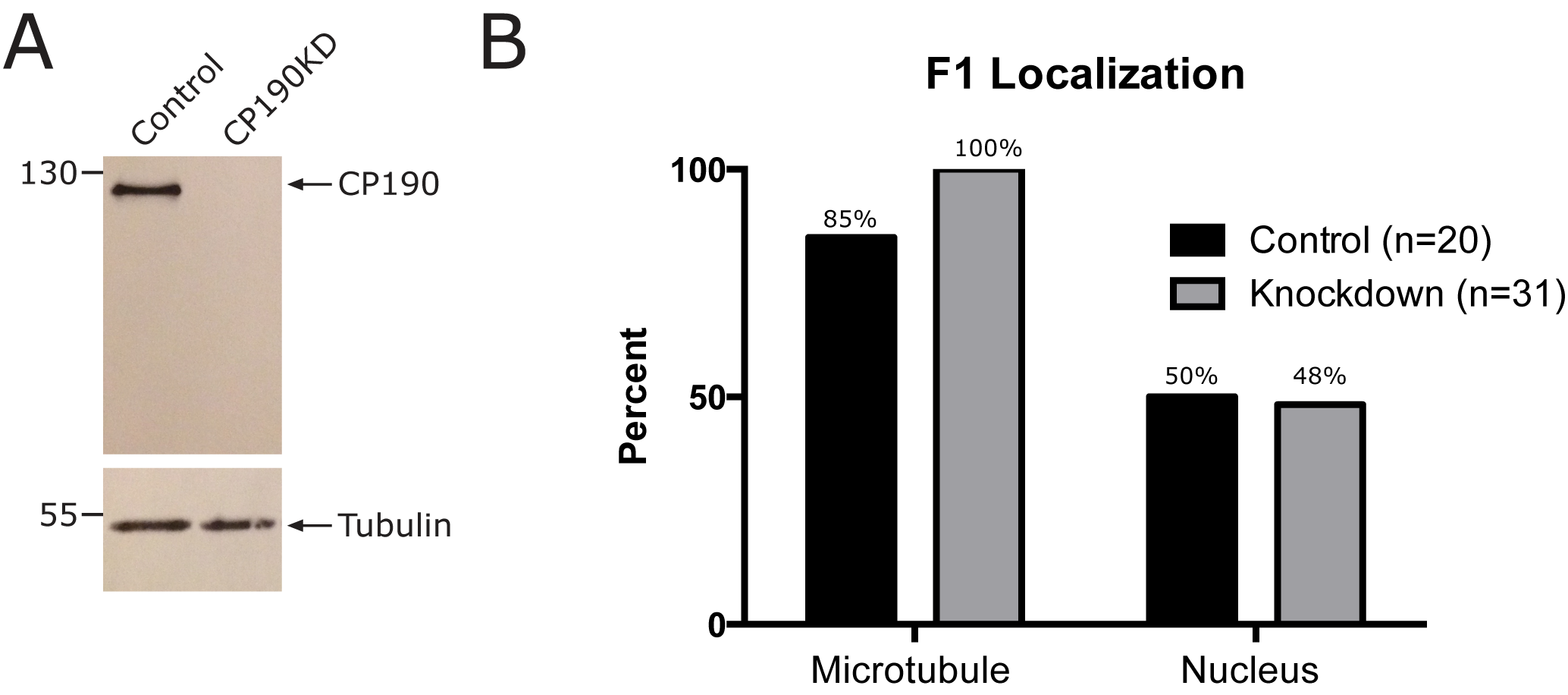

Supplement: S2 Fig — A. S2 cells were treated with dsRNA to knockdown endogenous CP190 and transfected with CP190 F1. Western blot shows effective CP190 knockdown (KD). A loading control (tubulin) is shown below. MW is indicated at left (kDa). B. Quantification of MT co-localization was completed in control and knockdown backgrounds using live cell imaging. The observed frequency of cells displaying CP190 F1 MT co-localization is on par with data presented in Fig 2B. We note, however, that the observed frequency of cells displaying CP190 F1 nuclear localization was significantly higher when analyzed using live cell imaging rather than fixed cell imaging. This difference is likely due to the harsh fixation protocol used. (TIF) [file pone.0144174.s002.tif]

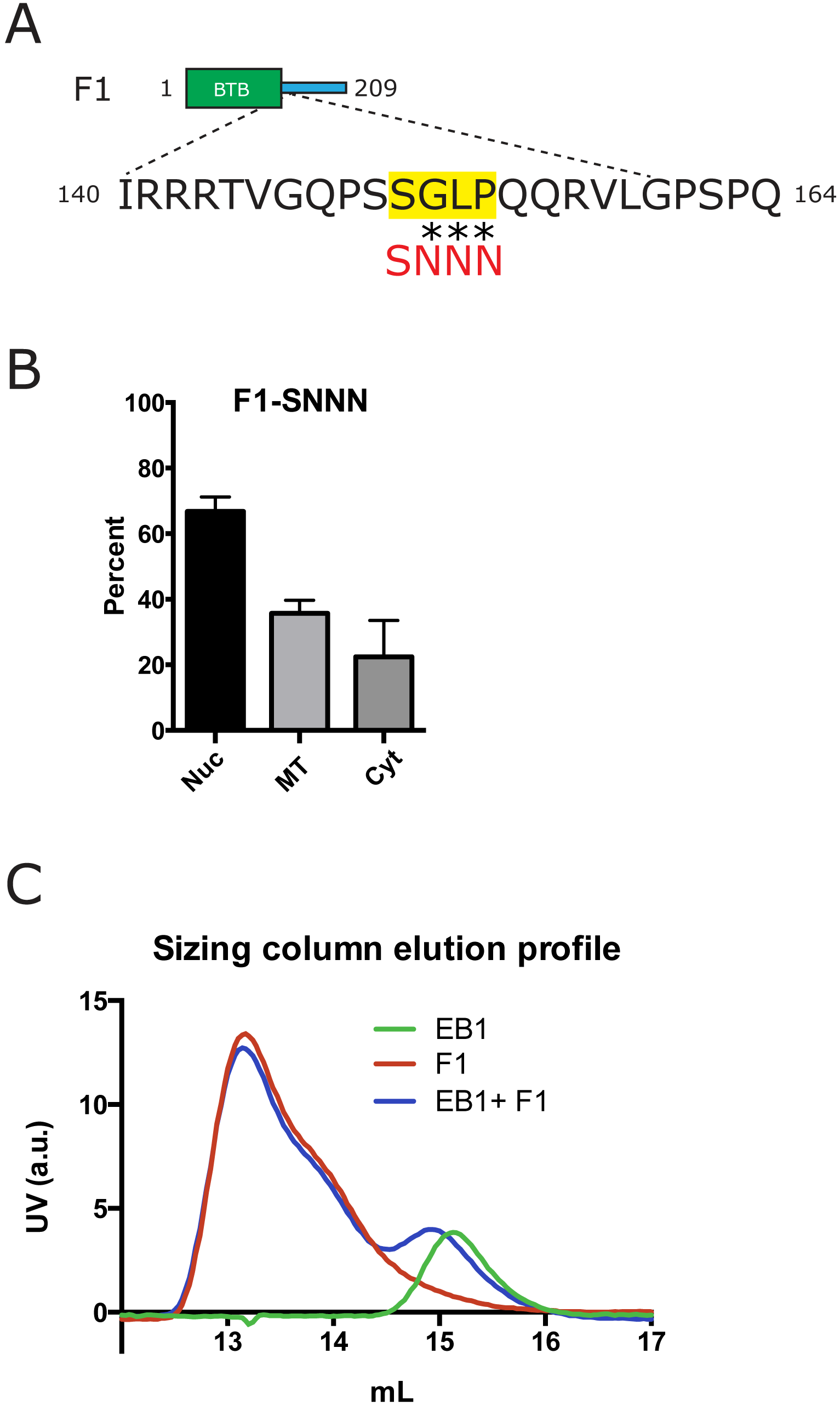

Supplement: S3 Fig — A, Analysis of CP190 F1 revealed an SxIP–like motif in the linker (F1-L) region (yellow). A F1 construct in which the SxIP-like motif residues were mutated to SNNN was analyzed using live-cell imaging. B, Cells were transfected with F1SNNN and TagRFP-Tubulin. Localization of CP190 GFP-F1SNNN to the nucleus, MTs, or diffuse localization the cytoplasm, was quantified. There is a significant reduction of CP190 GFP-F1SNNN as compared to WT F1 (Fig 2). C, Sizing column elution profile of EB1 (green), F1 (red), and EB1+F1 (blue). No significant peak shift is observed indicating that EB1 does not interact robustly with F1. (TIF) [file pone.0144174.s003.tif]

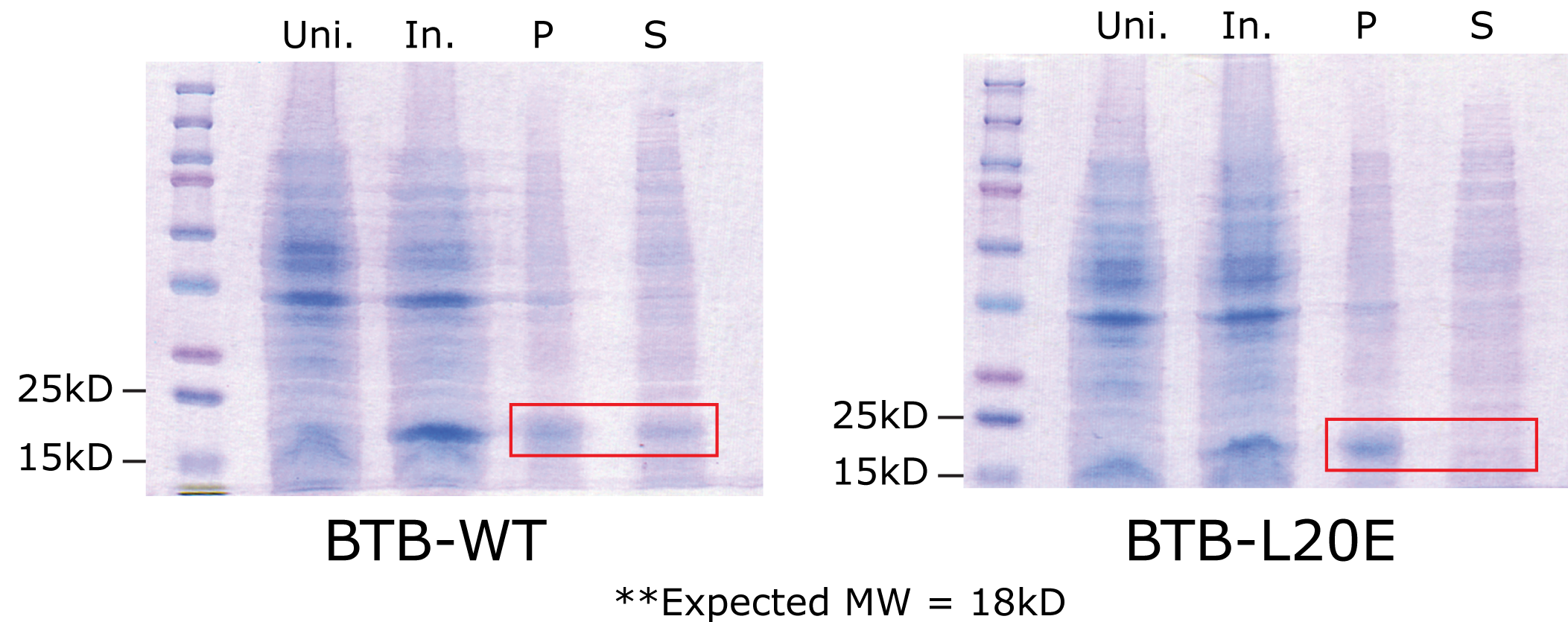

Supplement: S4 Fig — Mutating the BTB domain at the dimer interface renders the protein insoluble. Coomassie gels showing whole cell lysates from transformed E. coli growing the indicated protein: Uninduced (Uni.), Induced (In). Both BTB-WT and BTB L20E are produced in E. coli. Red boxes highlight insoluble protein in the pellet (P), and soluble protein in the supernatant (S) after lysis and centrifugation. BTB-WT has a soluble fraction, whereas BTB-L20E does not. (TIF) [file pone.0144174.s004.tif]

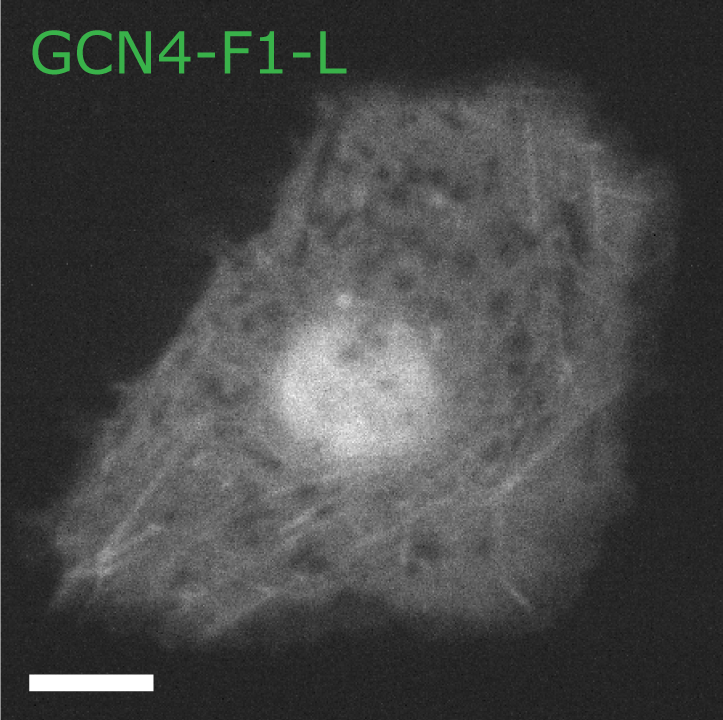

Supplement: S5 Fig — S2 cells transfected with The GCN4 dimerization domain fused to F1-L rescues MT localization activity, indicating that dimerization is important for localization to the MT lattice. Bar = 10 μm. (TIF) [file pone.0144174.s005.tif]

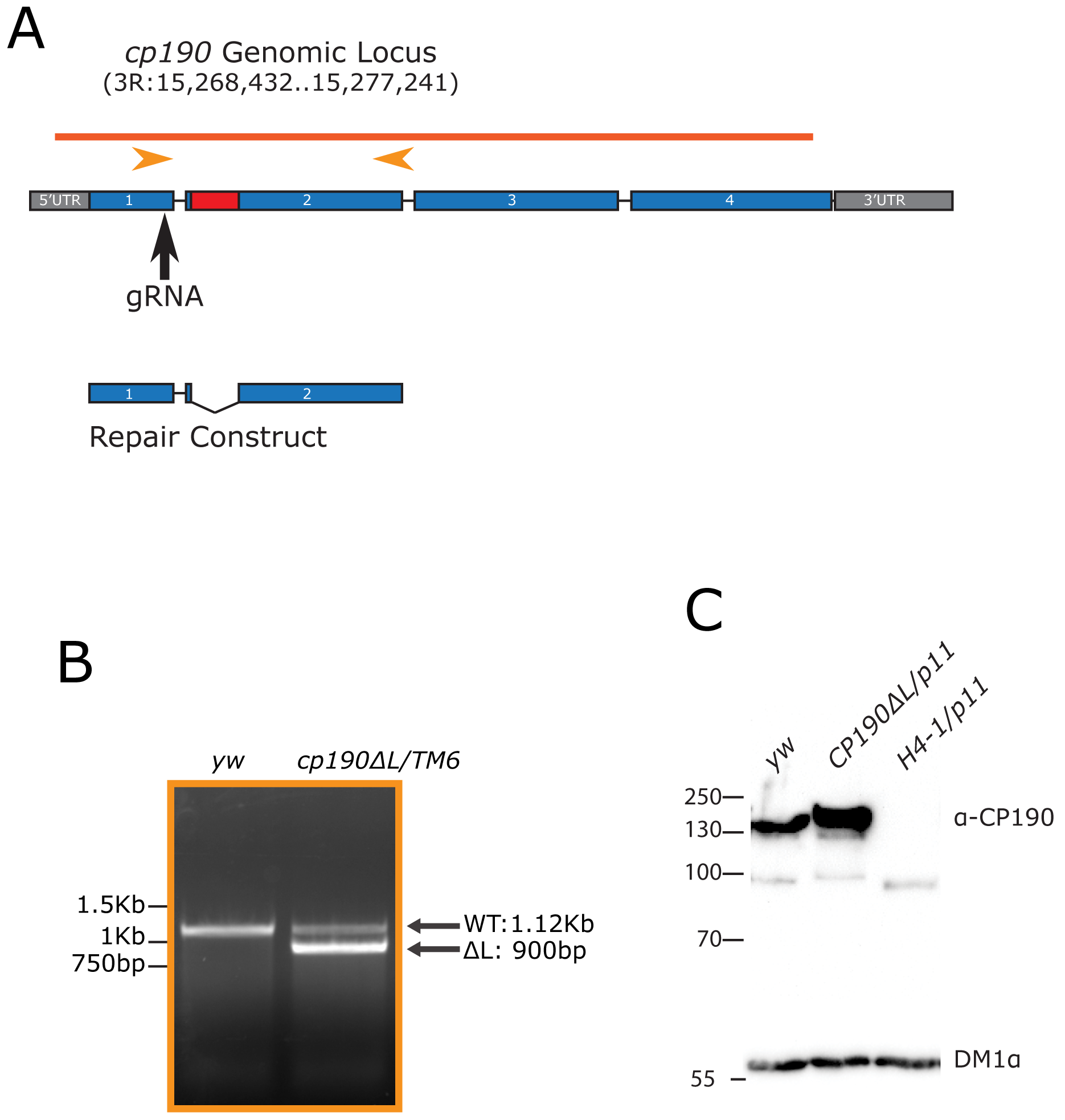

Supplement: S6 Fig — A. CP190 genomic locus. 5’ and 3’ UTR are in grey, exons are numbered in blue boxes, black lines indicated introns, red box in intron 2 is the linker region, PAM guide RNA site is indicated by black arrow. Below the genomic locus is a schematic of the repair construct. Primers used to screen for linker deletion in CRISPR flies are indicated in orange. Line above indicated sequence regions. B. PCR of control yw fly and a heterozygous cp190 ΔL fly. Expected sized are indicated to the right. C. Western blot shows that protein is produced in the cp190 ΔL fly. (TIF) [file pone.0144174.s006.tif]
